# Bandwidth Modeling of Silicon Retinas for Next Generation Visual Sensor Networks

**DOI:** 10.3390/s19081751

**Published:** 2019-04-12

**Authors:** Nabeel Khan, Maria G. Martini

**Affiliations:** Wireless and Multimedia Networking Research Group, Faculty of Science, Engineering and Computing, Kingston University, Penrhyn Rd, Kingston upon Thames KT1 2EE 1, UK; m.martini@kingston.ac.uk

**Keywords:** neuromorphic engineering, dynamic and active-pixel vision sensor, scene complexity, neuromorphic event rate, gradient approximation, scene texture, Sobel, Roberts, Prewitt

## Abstract

Silicon retinas, also known as Dynamic Vision Sensors (DVS) or event-based visual sensors, have shown great advantages in terms of low power consumption, low bandwidth, wide dynamic range and very high temporal resolution. Owing to such advantages as compared to conventional vision sensors, DVS devices are gaining more and more attention in various applications such as drone surveillance, robotics, high-speed motion photography, etc. The output of such sensors is a sequence of events rather than a series of frames as for classical cameras. Estimating the data rate of the stream of events associated with such sensors is needed for the appropriate design of transmission systems involving such sensors. In this work, we propose to consider information about the scene content and sensor speed to support such estimation, and we identify suitable metrics to quantify the complexity of the scene for this purpose. According to the results of this study, the event rate shows an exponential relationship with the metric associated with the complexity of the scene and linear relationships with the speed of the sensor. Based on these results, we propose a two-parameter model for the dependency of the event rate on scene complexity and sensor speed. The model achieves a prediction accuracy of approximately 88.4% for the outdoor environment along with the overall prediction performance of approximately 84%.

RMSE Root Mean Squared Error SSESum of Squares due to error DAVISDynamic and Active-pixel Vision Sensor IMU Inertial Measurement Unit CDF Cumulative Distribution Function DVS Dynamic Vision Sensor AER Address Event Representation MLE Maximum Likelihood Estimation

## 1. Introduction

Conventional video cameras capture video, via temporal sampling, in a series of separate frames or images, whose raw pixel values are then processed. In the conventional video sensing approach, the frames are typically acquired with a fixed frame rate, regardless of the scene content and complexity. This approach generates a substantial load in terms of energy consumption, data management and the transmission system. Furthermore, due to technological and resource limitations, temporal resolution is limited, and this may cause critical data loss between the captured frames. For instance, in a highly dynamic scene like an explosion, the fast-moving sections in the scene cause motion blur due to the frame rate and processing power restrictions. Therefore, frame-based video sensing does not appear as the best solution in terms of energy and efficiency for next-generation Internet of Things surveillance, autonomous driving/drone technology and robotics. The most efficient visual computing system is based on biological vision and perception in mammals, which is not based on pixels and frame-based sampling. The mammalian brain only receives new information from the eyes when something in a scene changes. This significantly reduces the amount of information delivered to the biological brain, but is sufficient to identify the surrounding changes and conditions.

The rest of this section discusses the Dynamic Vision Sensor (DVS) principle, the relevant literature of scene complexity metrics and the motivation of this study, followed by the contribution provided in this paper.

### 1.1. Dynamic Vision Sensors

Based on the principle of biological sensing, silicon retina devices capture only changes in scene reflectance, i.e., they report only the on/off triggering of brightness in the observed scene [1]. The first electronic model of retina was developed by [2], which comprises 700 photoreceptors and the same number of output cells. Later, the authors in [3] designed the first silicon model of retinal processing on a single silicon chip. The authors utilized photoreceptors that compute the logarithm of the incident light intensity. Recent developments in bio-inspired sensor systems [4,5] paved the way for event-driven DVSs.

A team led by Tobi Delbruck enhanced the neuromorphic silicon retina sensor based on the adaptive photoreceptor circuits concept [4]. Pixels in the 128 × 128 silicon retina sensor quantize the relative change in luminance intensity by generating spike events. These spike events output an asynchronous stream of digital addresses corresponding to the pixels with luminance intensity changes. This principle of neuromorphic vision sensors reduces redundant data since only brightness changes are transmitted, which not only reduces the amount of data processing, but also reduces latencies and brings temporal precision down to microseconds. In other words, there is a 10-fold reduction in power consumption, i.e., 10–20 mW of power requirements for DVS camera [4] as compared to hundreds of milliwatts of power consumption for conventional frame-based vision sensors. Furthermore, there is a 100-fold increase in speed, i.e., rendering events as frames can achieve a frame rate as high as 1000 frames per second [6]. These features, coupled with a wide dynamic range of 120 dB [4,7] compared to the 60-dB range for conventional vision sensors, makes these sensors highly desirable in applications such as real-time surveillance and tracking for robotics, drone technology, driverless cars, etc.

**Figure 1:**
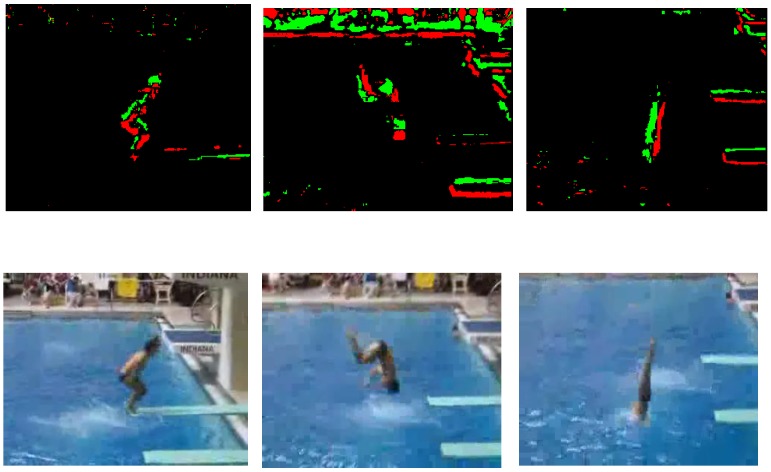
Top: neuromorphic vision sensor events rendered as frames. Bottom: conventional pixel frames.

Neuromorphic vision sensors are data driven, i.e., they asynchronously transmit events that are pixel-level relative intensity (brightness) changes. These events are produced with micro-second time resolution. The neuromorphic silicon technology utilizes the Address Event Representation (AER) protocol for exchanging data. The protocol, proposed by [8], is used as a communication method to transfer neuronal information within biological systems. AER is represented by a tuple (x,y,t,p), where *x* and *y* are the coordinates of the pixel that has undergone a brightness change, *t* is the timestamp and *p* is the polarity of the event. The polarity of the event represents either a positive, increase, or negative, decrease, sign of the grey-level intensity change. It is important to note that events are triggered whenever there is a movement of the neuromorphic vision sensor or motion in the scene or both. In other words, no data are transmitted for stationary vision sensors and static scenes. These unique properties enable neuromorphic vision sensors to achieve low-bandwidth, low-latency and low-power requirements. Figure 1 shows the conventional pixel frame and the neuromorphic sensor output. The first row of the figure shows the neuromorphic spike events, spatio-temporal coordinates {x,y,t} and polarity *p*, rendered as frames. The “green” colour represents events with positive change in brightness, whereas events with negative change in brightness are represented by the “red” colour.

In order to compare the bandwidth of the neuromorphic and conventional vision sensors, let us consider the data rate requirements of the “diving ” sequence with a spatial resolution of 320×240, as shown in Figure 1. The eight second-long sequence results in an average neuromorphic event rate of 28.67 Keps (kilo-events per second). According to [7,9], two bytes are required for transmitting an event. Therefore, the data rate of the diving sequence is approximately 57.34 kB/s (kilobytes per second). Furthermore, it is important to note that the data rate of 57.34 kB/s achieves a frame rate of 1000 fps. On the other hand, the data rate required to transmit raw video (grey-scale only), assuming 30 fps, is 320×240×30 B/s =2304 KB/s. This rate is about 40-times higher than the DVS data rate, and the temporal resolution of 1000 fps for DVS is 33.33-times higher than the conventional vision sensor.

### 1.2. Scene Complexity

In computer vision research, scene complexity information is useful in many applications, i.e., it can be used to determine the bandwidth requirement, as well as the amount of compression that can be tolerated. Furthermore, it can also support image clustering and classification. The definition of image complexity is widely varied, ranging from subjective complexity measures [10] to diverse objective metrics [11]. In computer vision research, scene complexity based on grey-scale images has been widely used, where an image-based metric is used as an objective measure. The spatial information of an image [12], as a measure of edge energy, is one of the most widely-used objective metrics. For instance, the work in [13] utilized scene complexity information in terms of spatial content of an image to predict the rate-distortion model of a video sequence. Kolmogorov introduced an algorithmic information theory-based complexity measure [14], which is defined as the amount of computational resources needed to produce an output. In other words, it is defined as the length of the shortest computer program that produces an objective output. However, the Kolmogorov complexity is not computable as there is no objective measure to define it. According to [11,15], approximate Kolmogorov complexity is the ratio between the size of uncompressed intensity image and the size of the compressed grey-scale intensity image. Another definition of image complexity is based on Shannon’s information theory concept called entropy. According to [14], the entropy of an image is a measure of uncertainty or the amount of information present in an image. Entropy is used extensively in determining the lower bound in the amount of lossless compression. In order to determine the complexity of an image, the authors in [16] proposed a fuzzy-based approach. The aim of the approach was to utilize an objective measure, entropy, and design a fuzzy-based mathematical model that fits to human perceived complexity. The authors in [17] proposed image complexity as a function of lossless compression, i.e., the complexity is defined as the inverse of the lossless compression ratio of the image. The authors in [18] defined image complexity as the ratio of lossy compression and distortion, where distortion is defined as the mean squared error between the original image and the compressed image. The aforementioned image complexity-based objective metrics have been used extensively in computer vision research for determining the level of compression, bandwidth allocation requirements and several other important tasks.

### 1.3. Motivation and Novelty

**Figure 2:**
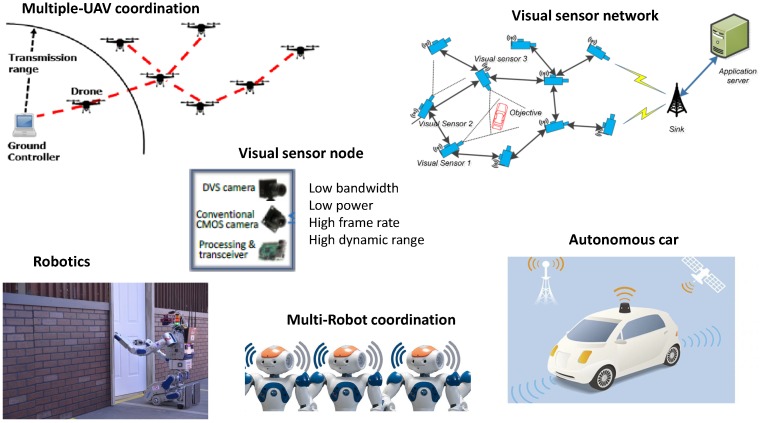
Key application scenarios of the Dynamic Vision Sensor (DVS).

Emerging applications of the DVS can be found in robotics [19,20], autonomic driving [21] and drones [22]. In the “Internet of Silicon Retinas ” (IoSiRe) project, we proposed a scenario where multiple neuromorphic vision sensors are interconnected and exchange the collected data. Figure 2 shows the key application scenarios of the neuromorphic vision sensors, where communication and coordination modules transmit the neuromorphic visual data. For instance, one of the key scenarios is the multiple coordinated Unmanned aerial Vehicle (UAV) system, where reliability and diversity are achieved by observing the area of interest from multiple points of view. In such a scenario, estimating the data rate output by the vision sensors is critical for the design of the communication strategies, such as scheduling and radio resource allocation [23,24,25,26]. Dynamic vision sensors provide high-frequency updates, corresponding to 1000 frames/s, as compared to standard cameras, where the maximum achieved frame rate is 60 frames/s. Therefore, another key application scenario of the DVS is high-speed multi-robot coordination, as shown in Figure 2.

The main aim of this work is to estimate the data rate resulting from DVS sensors. Since we expect the data rate will depend on the content of the scene, as anticipated in [27], one of our objectives was to identify content features (e.g., scene complexity) that can be used for the estimation of such a data rate.

In order to select the most appropriate one for the estimation of the data rate, the correlation of different scene complexity metrics with the neuromorphic event rate has to be investigated. According to the best of the authors’ knowledge, there has been no study done on the relationship between scene complexity metrics and neuromorphic event rate; hence, this represents the first novel contribution. The second important contribution is the identification of the relationship between the date rate and motion speed of the sensor. These two important contributions form the basis for the design of our proposed bandwidth model for neuromorphic vision sensors.

### 1.4. Contributions Provided in This Paper

This work aims at answering the following research questions:

- Which of the available scene complexity metrics shows the best correlation with the event rate?
- Can we define a novel scene complexity metric showing better correlation with the event rate?
- How can we model the relationship between the event rate and the scene complexity metric?
- How can we model the relationship between the event rate and the motion speed of the sensor?
- Finally, how can we model the neuromorphic data rate as a function of both the scene complexity metric and the motion speed of the sensor?

The remainder of this paper is structured as follows. Section 2 describes the adopted methodology. Section 3 describes the scene complexity metrics used and developed. The derived model for predicting the data rate for DVSs as a function of the scene complexity metric and motion speed is described in Section 4. Section 5 reports the data rate comparison between the neuromorphic and the conventional vision sensors. Finally, Section 6 concludes the paper.

## 2. Materials and Methods

### 2.1. Dataset

**Figure 3:**
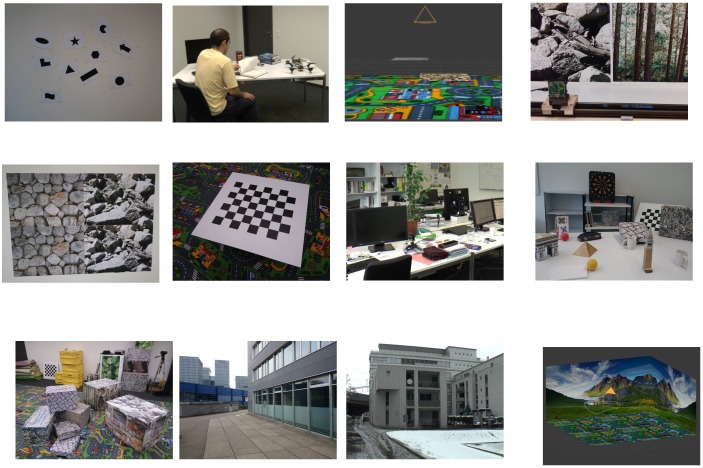
Different types of scenes in the Dynamic and Active-pixel Vision Sensor (DAVIS) dataset [28].

In order to model the event rate of neuromorphic vision sensors for different types of scenes, we utilized a publicly-available well-calibrated event-camera dataset [28]. The available dataset facilitates comparison and analysis of events’ generation, with information on the motion of the neuromorphic vision sensor. The dataset makes it possible to study and analyse spike event generation in relation with diverse types of scene complexity and varying degrees of camera motion. The dataset is available in two different formats, i.e., standard text form and binary rosbag files. In this work, we utilized the standard text format mainly because the associated files can be loaded easily using any high-level programming language such as Python or any computing environment such as MATLAB. The dataset was produced by utilizing a novel vision sensor technology known as Dynamic and Active-pixel Vision Sensor (DAVIS). DAVIS [7] is a hybrid sensor that combines the capabilities of classical frame-based vision sensors and of event-based neuromorphic vision sensors. The spatial resolution of the DAVIS camera is 240×180 pixels. A unique characteristic of the the DAVIS camera is the generation of frames and events by the same physical pixels. This makes it possible to output grey-scale intensity images (with each pixel represented by 8 bits, i.e., 0–255 grey scale levels) and neuromorphic event stream concurrently. In addition, DAVIS also includes an Inertial Measurement Unit (IMU), which provides information on the acceleration and velocity of the vision sensor in three dimensions. The position of the IMU was the same as that of the vision sensor, i.e., the IMU was mounted at only 3 mm behind the vision sensor, but centred under it; hence, the three dimension axes of the IMU were aligned with that of the vision sensor. The dataset comprises data for a variety of scenes ranging from indoors to outdoors to three-dimensional synthetic scenes, as shown in Figure 3. Furthermore, varying degrees of velocity of the sensor are considered.

The dataset contains the following important information:

- The asynchronous neuromorphic event stream in the form of the aforementioned tuple, i.e., (x,y,t,p).
- The frame-based output, in the form of intensity images, at approximately 24 frames per second. Each intensity image is precisely time stamped, which enables the recording of the number of neuromorphic events between any two intensity images.
- Inertial measurements in the form of a three-dimensional acceleration and velocity reported at a 1-ms interval. This information makes it possible to study the asynchronous event rate *w.r.t.* the three-dimensional velocity.

**Figure 4:**
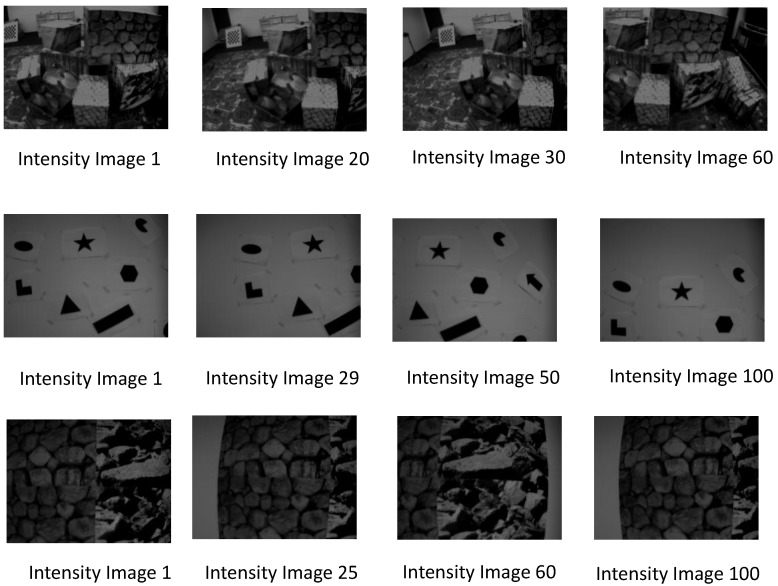
Intensity images at different velocities of the camera [28]. First row: Boxscene; Second row: Shapes scene; Third row: Poster scene.

**Figure 5:**
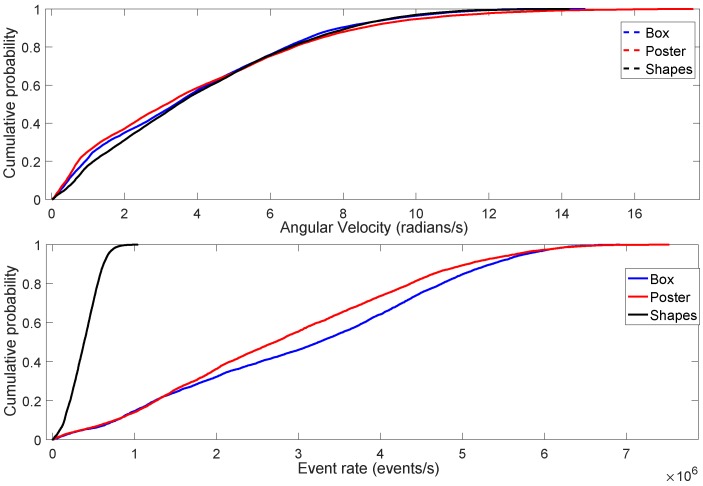
Top: CDF of the angular velocity of Box, Poster and Shapes scenes. Bottom: CDF of the neuromorphic event rate.

The aforementioned dataset allows us to study the relationship between the neuromorphic event rate and the type of scene. It is important to note that the neuromorphic bandwidth depends on the relative brightness change per-pixel, which depends on the pixels’ absolute intensity values. In other words, information on the distribution of pixels’ static light intensity, or brightness levels, in an image can be useful in determining the total number of the generated neuromorphic spike events. For instance, consider the three different types of scenes in Figure 4. According to the figure, the leftmost column shows the first intensity image from each of the three different types of scenes of the dataset. The motion of the DAVIS camera produces the subsequent intensity images, at approximately 24 frames per second, as shown in each of the three rows in Figure 4. Some pixels undergo intensity changes depending on the static intensity levels in a scene. Figure 5 shows the Cumulative Distribution Function (CDF) of the angular velocity and event rate. According to the figure, the angular velocity of DAVIS is approximately the same in all three scenes. However, the rate of change of brightness (event rate) of the *Shapes* scene is very low as compared to the *Box* and *Poster* scenes. This is mainly due to the fact that the variability among pixels’ absolute intensity levels is low in *Shapes* as compared to the *Box* and *Poster* scenes. In other words, the majority of the pixels of the *Shapes* scene have the same brightness levels; therefore, the motion of the camera triggers less brightness changes. On the other hand, the variability in the brightness levels of the *Poster* and *Box* scenes is very high, which translates into a higher neuromorphic event rate, as shown in Figure 5. Therefore, studying the relationship between type of scene and neuromorphic event rate is important to understand how the neuromorphic bandwidth varies with scene complexity.

### 2.2. Methodology

**Figure 6:**
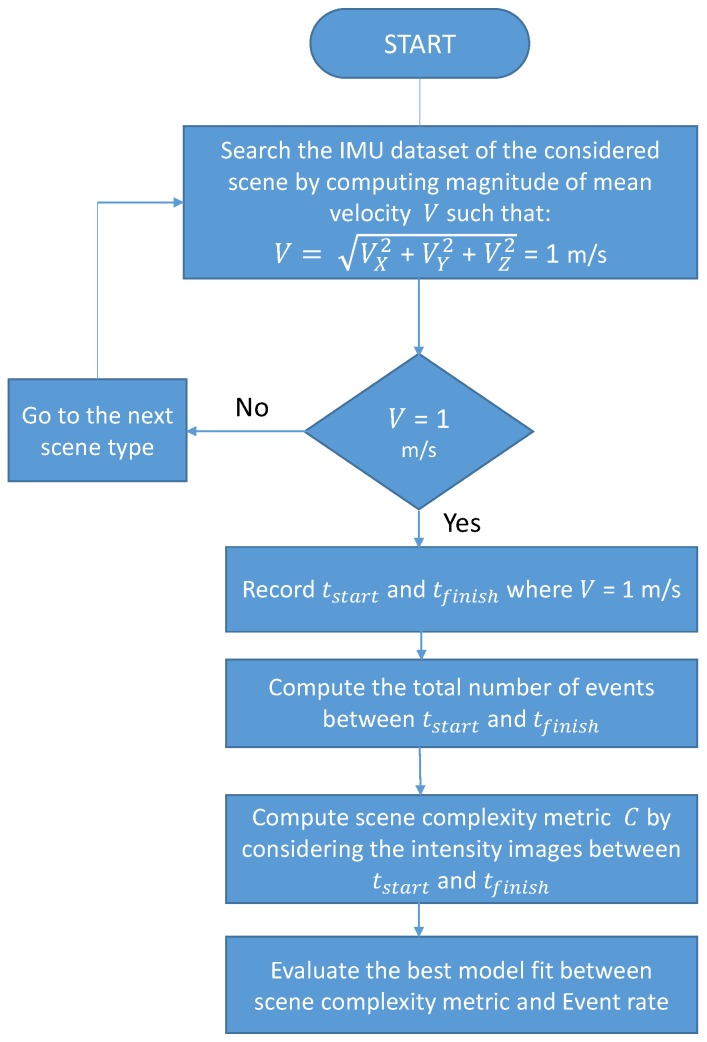
Adopted methodology for the correlation performance analysis between scene complexity and event rate.

In order to study and compare the correlation performance of different scene complexity metrics on the event rate, we considered similar motion speed of the vision sensor for every type of scene so that the event rate only depended on the scene complexity. Figure 6 shows the adopted methodology for the correlation performance analysis between scene complexity and event rate.

The adopted methodology is summarized in the following steps, with Figure 6 describing Steps 1–4.

1. Selection of data portions with similar speed of the capturing sensor: According to the analysis of the IMU dataset, the majority of the scene types comprised a magnitude of mean velocity of 1 m/s. Therefore, for every scene, we extracted the data (event rate and intensity images) such that the magnitude of mean velocity was 1 m/s. According to Figure 6, $t_{start}$ and $t_{finish}$ are recorded for the extracted dataset of a scene. The spike events and images between $t_{start}$ and $t_{finish}$ were utilized for the computation of event rate and scene complexity metrics.
2. Event rate computation: We computed the total number of neuromorphic events generated, $T_{\mathrm{events}}$, for each type of scene. Since different scene types have different time durations, we computed the event rate $T_{\mathrm{event}–\mathrm{rate}}$ by considering the duration, $T_{\mathrm{Duration}}=t_{finish}-t_{start}$, for every type of scene, i.e., $T_{\mathrm{event}–\mathrm{rate}}=\frac{T_{events}}{T_{\mathrm{Duration}}}$.
3. Scene complexity metric computation: The neuromorphic event stream and intensity images were produced concurrently by the DAVIS camera. In the dataset, intensity images were precisely timestamped; therefore, we computed the scene complexity metric by utilizing the intensity images between $t_{start}$ and $t_{finish}$.
4. Best model fit between scene complexity and event rate: Next, we found the best model (linear, exponential, power, etc.), for the relationship between scene complexity metric and event rate. Section 3 discusses the correlation performance of several scene complexity metrics *w.r.t* the neuromorphic event rate.
5. Best model fit between motion and event rate: Our study of the correlation between scene complexity and event rate initially assumed the same mean sensor speed for the different scenes. On the other hand, in order to analyse the correlation performance between motion speed and event rate, the value assumed by the scene complexity metric must remain constant so that the event rate varies as a function of the sensor motion speed only. Section 4.1 studies the impact of motion on the event rate.
6. In the final step, we propose a model for the neuromorphic event rate by utilizing the scene complexity metric and information on the motion of the camera (see Section 4.2).

## 3. Scene Complexity Metrics

The operation of dynamic vision sensors is based on the generation of spike events at local relative changes in brightness. Therefore, identifying and computing abrupt changes in pixel intensity in a scene becomes relevant for our study. In order to locate sharp discontinuities in an intensity image and study its impact on the neuromorphic event rate, the proposed scene complexity metrics range from gradient approximation to texture computation of an intensity image. In this section, we study the impact of such scene complexity metrics on the event rate. The details of these metrics, including their correlation performance with the neuromorphic event rate, are reported in the following subsections.

### 3.1. Metrics Based on Gradient Approximation of Intensity Images

The gradient of an image specifies the directional change in the intensity of an image. Furthermore, it also highlights the magnitude of significant transition in intensity. The main step in computing the gradient approximation of an image is to convolve the intensity image with a small finite filter known as the kernel. The filter is convolved with the intensity image in the horizontal and vertical directions. The three well-known methods to calculate such gradient are Sobel [29], Prewitt [30] and Roberts [31].

#### 3.1.1. Sobel Filter

The Sobel filtering process consists of convolving an intensity image with an integer valued filter. Let *I* denote an intensity image and $G^{x}$ and $G^{y}$ represent the horizontal and vertical gradients, respectively; the Sobel filtering process is formulated as:

(1) $$G^{x}=F_{x}*I$$

and:

(2) $$G^{y}=F_{y}*I$$

where $F_{x}$ and $F_{y}$ are 3×3 Sobel kernels for the horizontal and vertical directions, respectively, and are given as:

$$F_{x}=\left[\begin{array}{ccc} +1 & +2 & +1\\ 0 & 0 & 0\\ -1 & -2 & -1 \end{array}\right]$$

and:

$$F_{y}=\left[\begin{array}{ccc} +1 & 0 & -1\\ +2 & 0 & -2\\ +1 & 0 & -1 \end{array}\right].$$

The convolution is simply a linear combination of the kernel and the image. Each convolution operation results in a single gradient pixel output. Let $I^{\left(3\times 3\right)}$ be a 3×3 pixel intensity matrix:

(3) $$I^{\left(3\times 3\right)}=\left[\begin{array}{ccc} I_{i-1,j-1} & I_{i-1,j} & I_{i-1,j+1}\\ I_{i,j-1} & I_{i,j} & I_{i,j+1}\\ I_{i+1,j-1} & I_{i+1,j} & I_{i+1,j+1} \end{array}\right]$$

where i,j is the position of the pixel. The convolution process between $F_{x}$ and $I^{\left(3\times 3\right)}$ is a pixel-by-pixel multiplication. As the centre row of the kernel is zero, the gradient, $G_{i,j}^{x}$, is simply the difference in the weighted sum of pixel intensities between the first and third row.

(4) $$G_{i,j}^{x}=\left(I_{i-1,j-1}\cdot 1\right)+\left(I_{i-1,j}\cdot 2\right)+\left(I_{i-1,j+1}\cdot 1\right)+\left(I_{i+1,j-1}\cdot -1\right)+\left(I_{i+1,j}\cdot -2\right)+\left(I_{i+1,j+1}\cdot -1\right)$$

Similarly, the gradient, $G_{i,j}^{y}$, is the difference in the weighted sum of pixel intensities between the first and third column. The magnitude of the gradient is computed as:

(5) $$G_{i,j}=\sqrt{{\left(G_{i,j}^{x}\right)}^{2}+{\left(G_{i,j}^{y}\right)}^{2}}$$

where $G_{i,j}$ is the magnitude of the gradient of pixel $I_{i,j}$.

#### 3.1.2. Prewitt Filter

The Prewitt operator [30] is similar to the Sobel operator. Like Sobel, the Prewitt operator computes gradient approximation both in the horizontal and vertical directions. The derivative masks, the kernel, of the Prewitt operators are given as:

$$F_{x}=\left[\begin{array}{ccc} +1 & +1 & +1\\ 0 & 0 & 0\\ -1 & -1 & -1 \end{array}\right]$$

$$F_{y}=\left[\begin{array}{ccc} +1 & 0 & -1\\ +1 & 0 & -1\\ +1 & 0 & -1 \end{array}\right].$$

The convolution process of pixel-by-pixel multiplication is the same as that of the Sobel filter, as given by Equation (4). The major difference between the two operators is that the Sobel operator allocates more weights in the masks, which increases the magnitude of pixel intensities when the convolution operation is performed.

#### 3.1.3. Roberts Kernel

The Roberts operator [31] uses 2×2 derivative masks as compared to the 3×3 derivative masks of the Sobel and Prewitt operators. Furthermore, the first order derivative of the Roberts operator is computed in the diagonal directions. The derivative masks of the Roberts operator are:

$$F_{x}=\left[\begin{array}{cc} +1 & 0\\ 0 & -1 \end{array}\right]$$

$$F_{y}=\left[\begin{array}{cc} 0 & +1\\ -1 & 0 \end{array}\right].$$

The convolution operation is performed by using the 2×2 matrix of the intensity image as given below:

$$I^{\left(2\times 2\right)}=\left[\begin{array}{cc} I_{i,j} & I_{i,j+1}\\ I_{i+1,j} & I_{i+1,j+1} \end{array}\right].$$

The convolution process between the horizontal kernel and 2×2 intensity image results in:

(6) $$G_{i,j}^{x}=\left(I_{i,j}\cdot 1\right)+\left(I_{i+1,j+1}\cdot -1\right).$$

#### 3.1.4. Computation Process for Spatial Content Metrics

The computation process for the magnitude of the gradient is summarized in Algorithm 1. The first order derivative is computed for the centred pixel in the 3×3 intensity image. The derivative is computed for each of the pixels of the intensity image, i.e., 240×180 pixels gradient are computed. In order to compute the gradient of the border pixels, we utilized symmetric padding. In symmetric padding, padded pixels are the mirror reflection of the border pixels, i.e., the 3×3 intensity image is created by copying the intensity values of the border pixels. The output of the loop is the magnitude of the gradient image, as shown in Algorithm 1. In order to quantify the spatial information as a scene complexity metric, we computed the mean, standard deviation and root mean square of the gradient magnitude image *G*. These metrics were computed across all the pixels of the image. Let *M* and *N* be the number of rows and columns, respectively. The metrics are mathematically expressed as:

(7) $$SC_{\mathrm{mean}}=\frac{1}{M\cdot N}\sum_{j=1}^{N}\sum_{i=1}^{M}G_{i,j}$$

(8) $$SC_{\mathrm{rms}}=\sqrt{\frac{1}{M\cdot N}\sum_{j=1}^{N}\sum_{i=1}^{M}G_{i,j}^{2}}$$

and

(9) $$SC_{\mathrm{std}}=\sqrt{\frac{1}{M\cdot N}\sum_{j=1}^{N}\sum_{i=1}^{M}(G_{i,j}-SC_{\mathrm{mean}}{)}^{2}}$$

where $SC_{\mathrm{mean}}$, $SC_{\mathrm{rms}}$ and $SC_{\mathrm{std}}$ are the mean-, root mean square- and standard deviation-based spatial content metrics representing the edge energy of the intensity image *I*.

**Algorithm 1** Computation process for the magnitude of the gradient of an intensity image.Input: Intensity image $I^{M\cdot N}$ having M·N pixels**for**p=1 to M·N
**do** **if**
p==edgepixel
**then**  Use symmetric padding of the border pixel to create $I^{\left(3\times 3\right)}$ **else**  Construct a $I^{\left(3\times 3\right)}$ intensity matrix by considering a 3×3 neighbourhood around pixel *p* **end if** Compute the gradient for pixel *p* in the horizontal direction according to (4) Compute the gradient for pixel *p* in the vertical direction Compute the magnitude of the gradient for pixel *p* according to (5)
**end for**
Output: Gradient magnitude image *G*.

#### 3.1.5. Correlation Performance Between Neuromorphic Event Rate and Spatial Content Metrics

**Figure 7:**
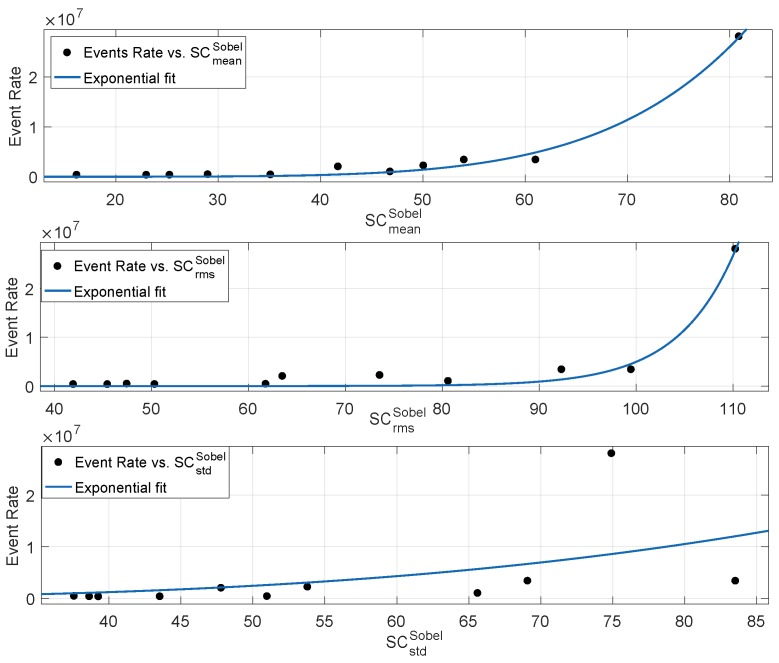
Relationship between event rate and spatial content metrics based on Sobel filtering.

In order to study the correlation between neuromorphic event rate and scene complexity metrics, we computed the mean, standard deviation and root mean square metrics based on the Sobel filter. The relationship between event rate and scene complexity metrics is shown in Figure 7, where the spatial content metrics of eleven diverse types of scenes are plotted against the event rate according to the methodology reported in Figure 6. According to Figure 7, the spatial metric based on the mean of the gradient shows the best correlation as compared to the standard deviation and root mean square-based metrics. The standard deviation of Sobel-based gradient approximation has been used extensively [32,33,34] in defining scene complexity for normal, as well as gaming videos. It is important to note that neuromorphic sensor motion triggers events based on relative-brightness changes. The mean of the gradient represents the per pixel average rate of change of brightness. Therefore, the higher the pixel average rate of change of intensity, $SC_{\mathrm{mean}}^{Sobel}$, in a scene, the higher the neuromorphic event rate. Table 1 reports the goodness of fit statistics in terms of $R^{2}$, adjusted $R^{2}$, Root Mean Squared Error (RMSE) and Sum of Squares due to error (SSE). Goodness of fit statistics $R^{2}$ and adjusted $R^{2}$ close to one represent a higher degree of correlation between the response values and fitted curve model. On the other hand, the higher the RMSE and SSE, the lower the degree of correlation. According to Table 1, among the Sobel-based metrics, the single-term exponential model showed the best fit between the neuromorphic event rate and mean pixel gradient $SC_{\mathrm{mean}}^{Sobel}$. On the other hand, the spatial content metric based on the standard deviation showed the worst goodness of fit statistics among the Sobel-based metrics.

**Figure 8:**
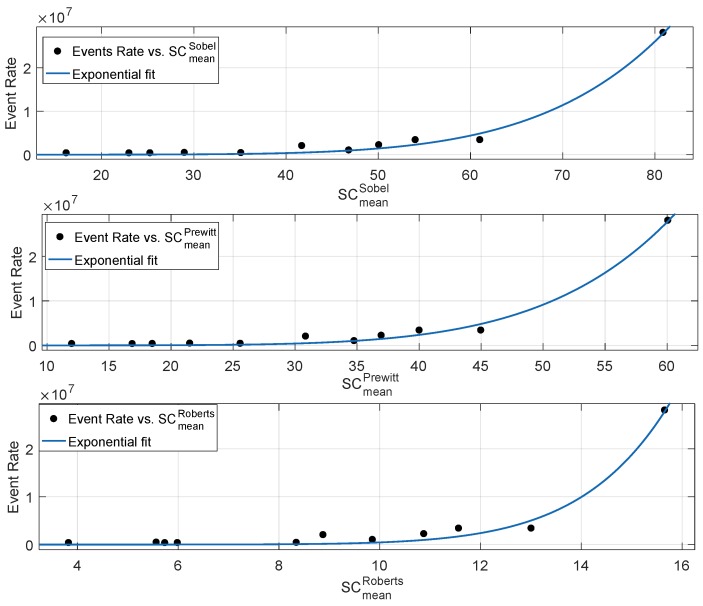
Relationship between neuromorphic event rate and mean gradient metrics based on Sobel, Prewitt and Roberts operators.

According to the aforementioned analysis, the spatial content metric $SC_{\mathrm{mean}}^{Sobel}$ showed the best correlation with the event rate. A similar behaviour was observed for Prewitt and Roberts filtering, where the mean of the gradient magnitude outperformed the standard deviation and root mean square-based spatial content metrics. Therefore, we computed the mean gradient metric based on Prewitt and Roberts operators and compared it with the Sobel-based mean spatial content metric, as shown in Figure 8. Like Sobel, Prewitt used a 3×3 kernel masks in the horizontal and vertical directions, i.e., both the operators detect the rate of change of intensity in the horizontal and vertical directions. Sobel kernels give more weights to the neighbouring pixels in the horizontal and the vertical directions, which enhances the difference in pixel intensities. Therefore, the magnitude of the gradient for the Sobel operator is higher than that of the Prewitt operator, as shown in Figure 8. It is important to note that mean gradient approximations based on Sobel and Prewitt have approximately the same performance in terms of correlation with the event rate as shown in Table 1. This is mainly because both the operators have the same working operation, i.e., both the operators highlight prominent horizontal and vertical edges. This causes both the operators to detect even a minimal change of brightness around the neighbouring pixels. This feature highly correlates with the event stream production principle of neuromorphic sensors, where a small local level intensity change in a pixel produces an event.

The key role of the Roberts operator is to detect intensity changes in diagonally-adjacent pixels by utilizing 2×2 kernels. The magnitude of the mean gradient of Roberts operator was the lowest as compared to the Sobel and Prewitt operators, as shown in Figure 8. According to Table 1, the Roberts operator showed the lowest correlation performance with the neuromorphic event rate among the considered mean gradient metrics. The main reason for a higher root mean square error for the Roberts-based mean gradient is the small size kernel’s susceptibility to noise. It is important to note that edge detection identifies pixels in a scene where brightness changes sharply. The higher the contribution of edge detection pixels to the mean gradient, the higher the correlation performance *w.r.t* the neuromorphic event rate. The small size Roberts kernel produced weak response to genuine edges, i.e., the set of points where scene brightness changes abruptly is affected by noise. The primary advantage of the Roberts operator is very low computational complexity as compared to the Sobel and Prewitt operators. Gradient based on horizontal and vertical directions requires eight neighbouring pixels, whereas gradient based on the Roberts cross operator requires only four neighbouring pixel, thus reducing the computation burden. However, with a kernel size of 2×2, pixels with a sharp rate of change of brightness contribute less to the mean gradient. Among the three considered kernels, Sobel and Prewitt operators showed the best correlation with the neuromorphic event rate followed by the Roberts operator, as shown in Table 1.

### 3.2. Metrics Based on Binary Edge Detection

The aforementioned metrics compute gradient approximation by utilizing discrete differential operators. The set of points where image brightness change abruptly is transformed into a set of curved line segments referred to as visual edges. By utilizing some common algorithms, pixels with a strong gradient magnitude are chained together to form a complete description of an edge. These algorithms extract different features by constraining different properties of an edge, such as gradient orientation or gradient magnitude thresholding. In this section, we investigate different gradient magnitude thresholding algorithms and study their correlation performance *w.r.t* the neuromorphic bandwidth. These algorithms include a variety of methods that convert an intensity image into a binary image, where a pixel with one indicates the presence of an edge and a pixel with zero represents the absence of an edge [35]. It is important to note that a higher number of edge pixels corresponds to a higher variation in scene illumination, in particular in the case that the sensor is not in a fixed position. Therefore, the higher the number of edge pixels, the higher will be the number of events generated by local brightness change due to the motion of the neuromorphic sensor. We study the following well-known binary edge detection methods.

#### 3.2.1. Edge Detection Based on a Single Threshold

Sobel, Prewitt and Roberts edge detection methods are based on the approximation of the gradient-based operation, as reported in Section 3.1. The first step of the binary edge detection step consists of converting an intensity image *I* into a gradient image *G* by utilizing either the Sobel, Prewitt or Roberts filtering process. After the approximation of the first derivative of the intensity image, the next step converts the gradient image into a binary image by using a specific gradient magnitude threshold. Pixels having a gradient magnitude greater than the threshold are assigned one. These are the pixels responsible for generating the maximum number of spike events. In order to measure the impact of binary edge detection on the neuromorphic event rate, we propose a metric based on the number of edge pixels. It is mathematically expressed as:

(10) $$\mathrm{EP}=\frac{1}{M\cdot N}\sum_{j=1}^{N}\sum_{i=1}^{M}B_{i,j}$$

with:

(11) $$B_{i,j}=\left\{\begin{array}{cc} 1, & \mathrm{For}\;\mathrm{all}\;\mathrm{edge}\;\mathrm{pixels}\\ 0, & \mathrm{otherwise}. \end{array}\right.$$

where EP is the proportion of edge pixels in binary image $B_{i,j}$, i.e., it is the ratio between edge pixels and the total number of pixels in the image. The binary image $B_{i,j}$ is either based on Sobel-, Prewitt- or Roberts-based gradient approximation.

#### 3.2.2. Edge Detection Based on a Dual Threshold

The Canny edge detection algorithm [36] is based on a dual threshold. It is the most efficient edge detection algorithm because of its capability to detect a wide range of edges. However, the Canny edge detection algorithm suffers from high computational complexity. The main steps involved in the Canny edge detection process are:

- Applying the Gaussian filter to the intensity image. The main advantage of the filter is smoothing the image by removing any noise present in the image.
- Computation of the gradient magnitude image *G* based on the Sobel filter. This step is similar to the process reported in Section 3.1.
- The next step is to enhance the gradient approximation image by a process known as non-maxima suppression. This process enhances the gradient magnitude of all the pixels by further reducing the noise. The output of this step is a filtered pixel gradient.
- The last step is a dual threshold action known as hysteresis. In this step, a filtered pixel gradient higher than the upper threshold is marked as an edge pixel. On the other hand, a pixel gradient lower than the lower threshold is marked with zero. If the gradient is in between the two thresholds, then the pixel is marked as an edge pixel only when the neighbouring connected pixel’s gradient is above the higher threshold.

By computing the percentage of binary edge pixels as reported in (10), we study the correlation between the index obtained via Canny edge detection and the neuromorphic event rate.

#### 3.2.3. Correlation Performance Between Neuromorphic Event Rate and Binary Edge-Based Metrics

**Figure 9:**
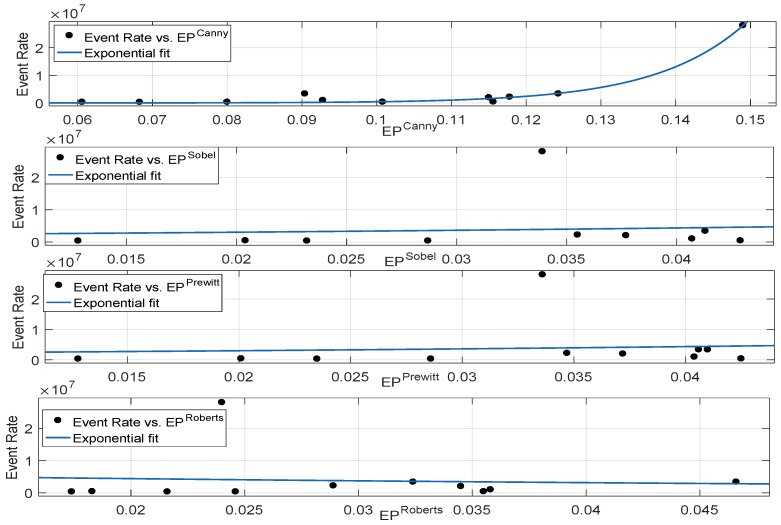
Relationship between neuromorphic event rate and binary edge detection metrics based on single and dual threshold (Canny) algorithms.

In order to study the correlation performance of the aforementioned metrics, we computed the Sobel-, Prewitt-, Roberts- and Canny-based binary edge pixels and studied their correlation performance with the neuromorphic event rate. According to Figure 9, Sobel-, Prewitt- and Roberts-based edge detection metrics show poor correlation performance. This is mainly because noise present in the image highly degrades the edge detection process. In other words, the single threshold-based process fails to identify all the edge pixels, and furthermore, it is sensitive to the noise present in the image. In neuromorphic vision sensors, each pixel can potentially be a source of event generation, and failure to identify edge pixels corresponds to a poor correlation performance with the event rate. The Canny edge detection process reduces the impact of noise by utilizing the Gaussian filter and non-maxima suppression steps. Furthermore, the dual threshold action improves the edge detection process and shows a good correlation performance. Table 2 reports the goodness of fit statistics for the exponential fit of all the considered binary edge-based metrics. Canny edge detection showed better correlation with the neuromorphic event rate; however, its performance in comparison with the mean gradient metrics was poor. For instance, the RMSE was approximately doubled for the Canny when compared to the performance of the Sobel and Prewitt-based mean gradient metrics (reported in Table 1). According to Table 2, single threshold-based binary edge detection of intensity images, captured with DAVIS frames, suffered from noise and failed to detect correctly all the edge pixels. The Canny method reduced the impact of noise as compared to other edge detection process and detected the majority of the true edges, but its correlation performance with the event rate was still not comparable to the mean gradient-based metrics analysed in Section 3.1.5. Therefore, compared to binary edge detection metrics, computation of the mean of the gradient magnitude, i.e., $SC_{mean}^{Sobel}$ and $SC_{mean}^{Prewitt}$, correlates better for neuromorphic vision sensors.

### 3.3. Metrics Based on Scene Texture

Spatial content based metrics, reported in Section 3.1, compute brightness variations among neighbouring pixels by utilizing finite integer filters. The higher the local variability among the neighbouring pixels, the higher the event rate. These metrics compute variability by computing the maximum rate of change of intensity in the horizontal and vertical directions. In this section, we propose three metrics that are also based on the variations in pixels intensities; but instead of computing gradient approximation, we utilized basic statistical measures such as standard deviation, range and entropy. For instance, consider the $I^{\left(3\times 3\right)}$ intensity matrix centred around pixel $I_{i,j}$ as reported in Equation (3); we propose to assign a value to the centred pixel based on the variability among the eight neighbouring pixels intensity values. Let *A* be a set of all eight neighbouring pixels around $I_{i,j}$ in the $I^{\left(3\times 3\right)}$ intensity matrix given in Equation (3).

(12) $$A=\{I_{i-1,j-1},\;I_{i-1,j},\;I_{i-1,j+1},\;I_{i,j-1},\;I_{i,j+1},\;I_{i+1,j-1},\;I_{i+1,j},\;I_{i+1,j+1}\}$$

Range intensity, $R_{i,j}$, for pixel $I_{i,j}$ is computed by considering the maximum and minimum intensity levels in *A*:

(13) $$R_{i,j}=max\left(A\right)-min\left(A\right)$$

Next, we compute the standard deviation by utilizing the intensity levels of the eight neighbouring pixels. The standard deviation for pixel $I_{i,j}$ is computed as:

(14) $$Std_{i,j}=\sqrt{\frac{1}{8}\sum_{n=1}^{8}(A_{n}-\bar{A}{)}^{2}}$$

where $\bar{A}$ is the mean intensity level of the eight neighbouring pixels, and entropy is computed by:

(15) $$E_{i,j}=\sum_{n=1}^{256}h_{n}\cdot \frac{1}{\log_{2}h_{n}}$$

where *h* is the normalized histogram counts, which is 256 bins for a grey-scale image. In other words, the histogram computation bins the intensity levels of eight neighbouring pixels into 256 equally-spaced containers. It is important to note that the entropy is computed by considering only the non-zero normalized histogram count, i.e., h≠0.

In order to compute the complexity metrics of the whole image, we compute the mean of the range, standard deviation and entropy as reported below:

(16) $$RI=\frac{1}{M\cdot N}\sum_{j=1}^{N}\sum_{i=1}^{M}R_{i,j}$$

(17) $$stdI=\frac{1}{M\cdot N}\sum_{j=1}^{N}\sum_{i=1}^{M}Std_{i,j}$$

(18) $$EI=\frac{1}{M\cdot N}\sum_{j=1}^{N}\sum_{i=1}^{M}E_{i,j}$$

where RI, stdI and EI are the range, standard deviation and entropy indexes of the intensity image I.

The higher the variability in terms of these statistical measures, the higher the roughness or bumpiness in the image texture. On the contrary, lower variations among the pixel intensities indicate smooth texture content. Algorithm 2 shows the computation process of the three proposed metrics. The computational complexity of the texture-based metrics is low as compared to the gradient approximation metrics. The texture-based metrics require two main computation steps, i.e., the first step is the per pixel computation of either the range or standard deviation or entropy. The second step is the computation of the mean of the statistical measure. On the other hand, gradient computation process, reported in Algorithm 1, requires four computation steps. For instance, consider the Sobel-based mean gradient computations. The first two steps comprise a per pixel rate of change of intensity in the horizontal and vertical directions. The third step is the computation of the gradient magnitude of each pixel according to Equation (5), whereas the final computation step is the calculation of the mean gradient by considering the magnitude of each pixel. In the subsequent section, we analyse the correlation performance of the texture-based metrics.

**Algorithm 2** Computation process of range, entropy and standard deviation indexes of the intensity image.Input: Intensity image $I^{M\cdot N}$ having M·N pixels**for**p=1 to M·N
**do** **if**
p==edgepixel
**then**  Use symmetric padding of the border pixel to create $I_{3\times 3}$ **else**  Construct an $I^{\left(3\times 3\right)}$ intensity matrix by considering a 3×3 neighbourhood around pixel *p* **end if** Compute the range of pixel *p* according to (13) Compute the standard deviation of pixel *p* according to (14) Compute the entropy of pixel *p* according to (15)
**end for**
Output: $R^{M\cdot N}$, $Std^{M\cdot N}$ and $E^{M\cdot N}$.Compute the range index according to (16)Compute the standard deviation index according to (17)Compute the entropy index according to (18)

#### Correlation Performance of Metrics Based on Scene Texture

**Figure 10:**
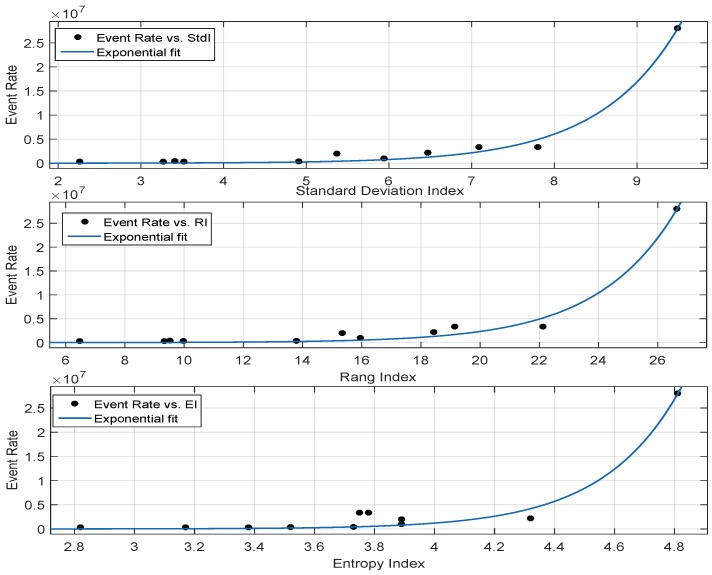
Exponential curve fitting of the range-, standard deviation- and entropy-based metrics.

The correlation performance of the range, standard deviation and entropy indexes is shown in Figure 10, whereas the goodness of fit statistics are reported in Table 3. According to the table, metrics based on range and standard deviation exhibited good correlation with the neuromorphic event rate, as illustrated by a lower RMSE. On the other hand, the RMSE performance for EI was higher as compared to the range- and standard deviation-based metrics. Both RI and stdI are a function of local variability in brightness among the neighbouring pixels, i.e., both the metrics quantify the per-pixel variations in terms of intensity levels. The higher the contrast among the neighbouring pixels, the higher the probability of a spike event. Conversely, image having large runs of pixels with the same brightness levels triggered a lesser number of spike events. EI exhibited a higher RMSE mainly because it does not take into account the spatial contrast variations of pixels. Furthermore, entropy is a function of the image intensity histogram, which is oblivious to the spatial structure of an image. Therefore, two contrasting images can result in having the same entropy because the per-pixel spatial heterogeneity is not considered. According to the goodness of fit statistics reported in Table 3, the exponential fit showed good correlation between the event rate- and the texture-based metrics. It is important to note that the Sobel- and Prewitt-based mean gradients showed better correlation as compared to the range and standard deviation indexes. However, the computational complexity of the range- and standard deviation-based metrics was lower as compared to the gradient-based metrics.

According to the detailed analysis, mean gradient metrics ($SC_{\mathrm{mean}}^{\mathrm{Sobel}}$, $SC_{\mathrm{mean}}^{\mathrm{Prewitt}}$ and $SC_{\mathrm{mean}}^{\mathrm{Roberts}}$) and texture-based metrics (RI, stdI) showed good correlation performance *w.r.t* the event rate. In the subsequent sections, we develop a neuromorphic bandwidth model based on the aforementioned best-performing scene complexity metrics.

## 4. Neuromorphic Data-Rate Model

The required bandwidth of the neuromorphic vision sensors depends on the scene content and speed of the camera. According to the correlation performance analysed in Section 3, there is an exponential relationship between the event rate and different scene complexity metrics. In order to design a bandwidth model of the neuromorphic vision sensors based on these metrics, the impact of the motion of the sensor on the neuromorphic event rate is analysed in the subsequent section.

### 4.1. Impact of Motion on Event Rate

#### 4.1.1. Indoor Environment

**Figure 11:**
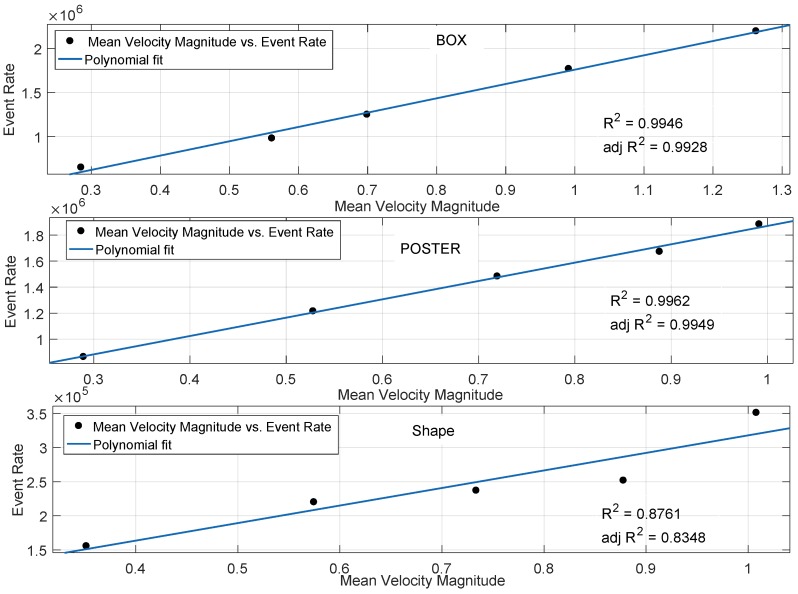
Correlation performance between neuromorphic event rate and magnitude of mean velocity by considering the Box, Poster and Shapes datasets.

To study the impact of motion on the event rate, we first utilized the Indoor datasets of *Shapes*, *Box* and *Poster*. The main rationale of using the Indoor dataset is the availability of static scene content. In other words, the mean gradient of the scene remains approximately constant over time, whereas the motion of the camera varies. Therefore, for the indoor dataset, the camera motion changes the event rate as the scene content remains approximately constant. This is in contrast to the outdoor dataset, where the scene content, as well as the motion of the camera impact the event rate. Therefore, the Indoor dataset allows us to analyse the relationship between camera motion and event rate generation. In the *Box*, *Shapes* and *Poster* datasets, the motion is initiated with the excitation of a single degree of freedom followed by faster excitations of three degrees of freedom. This leads to a higher event rate over time. Figure 11 reports the scatter plots between neuromorphic event rate and mean velocity. According to the figure, the relationship between camera motion and event rate is approximated on a first degree polynomial fit. The figure also reports the goodness of fit statistics in terms of $R^{2}$ and adjusted $R^{2}$. The polynomial fit shows an excellent correlation performance between the even rate and mean velocity for the *Box* and *Poster* scenes. The low complexity *Shapes* scene also shows a reasonable correlation performance. The linear polynomial relationship, between event rate and mean velocity, held true for different indoor scene content ranging from the low complexity *Shapes* scene to the more complex *Box* and *Poster* scenes.

#### 4.1.2. Outdoor Environment

**Figure 12:**
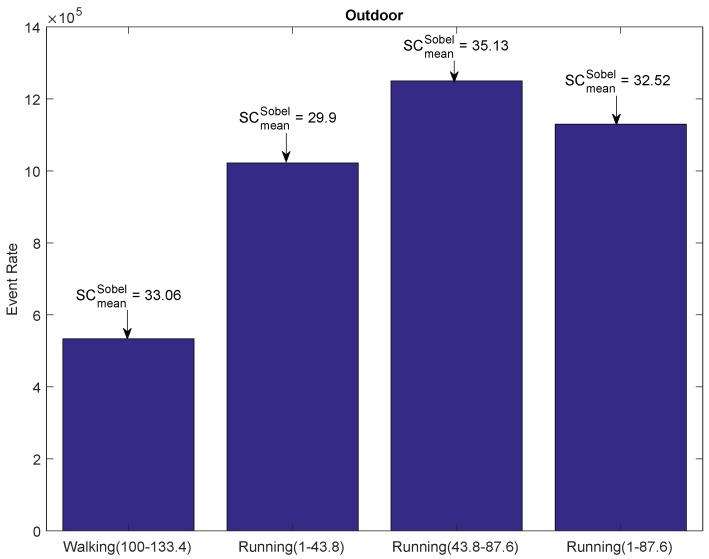
Event rate plot of the extracted sequences from the outdoor data. The text arrows report the mean gradient of the extracted sequences.

In order to study the impact of motion in the outdoor environment, we evaluated the outdoor *Walking* and *Running* dataset in [28]. In the outdoor environment, the scene content changes continuously during walking and running motion. The *Walking* dataset comprises a 133.4 second-long sequence of outdoor walking captured with a handheld DAVIS camera, whereas the duration for the *Running* dataset is 87.6 s. The average mean velocity for the *Walking* and *Running* dataset is 1.42 m/s and 3.1 m/s, respectively. Our main goal was to extract sequences from the two outdoor datasets, such that the resulting subsequences would have approximately the same scene complexity (mean gradient), but with different average sensor speed. The bar graphs in Figure 12 report such extracted sequences from the *Walking* and *Running* dataset. The text arrow above the bar graph shows the scene content in terms of Sobel-based mean gradient. The mean gradient varied widely within each dataset; however, the mean gradient for the 33.4-second walking sequence (Walking (100–133.4)) was very close to the 87.6 second-long sequence of the *Running* dataset (Running (1–87.6)). We observed the impact of motion on the event rate for the two sequences having approximately the same mean gradient; an increase in motion of 118% (1.42–3.1 m/s) increased the event rate by 112% (0.533–1.13 mega-events). Therefore, the relationship between the sensor speed and event rate for the outdoor environment can also be approximated by a linear function of mean velocity.

### 4.2. Model Formulation

In order to model the neuromorphic event rate as a function of scene complexity and motion, we propose to utilize a two-parameter single-term exponential model, where the event rate varies linearly with the magnitude of mean velocity *V* of the sensor and exponentially with the scene complexity metric *C*. The mean event rate model, μ, is formulated as:

(19) $$\mu =V\cdot a\cdot \exp\left(C\cdot b\right)$$

where *a* and *b* are the two parameters of the model. Section 3 analysed several scene complexity metrics. According to Table 1, mean gradient metrics (based on Sobel, Prewitt and Roberts) showed good correlation performance. Furthermore, the texture-based metrics RI, stdI and EI also performed well according to the goodness of fit statistics reported in Table 3.

In order to compute the two parameters, we utilized the training data in Figure 8 and Figure 10, where scene complexity metrics are plotted against the event rate with mean velocity kept constant at 1 m/s. We utilized the non-linear least squares method to compute the two parameters. The mean rate, μ, based on the Sobel-, Prewitt- and Roberts-based mean gradient can be written as:

$$\mu =\left\{\begin{array}{cc} V\cdot a\cdot \exp\left(SC_{\mathrm{mean}}^{\mathrm{Sobel}}\cdot b\right) & :a=1.84\times 10^{4},b=0.09055\\ V\cdot a\cdot \exp\left(SC_{\mathrm{mean}}^{\mathrm{Roberts}}\cdot b\right) & :a=1153,b=0.6446\\ V\cdot a\cdot \exp\left(SC_{\mathrm{mean}}^{\mathrm{Prewitt}}\cdot b\right) & :a=2.035\times 10^{4},b=0.1203 \end{array}\right.$$

The magnitude of mean velocity V of the sensor is given as:

(20) $$V=\sqrt{{V_{x}}^{2}+{V_{y}}^{2}+{V_{x}}^{2}}.$$

Similarly, the mean rate model based on RI, stdI and EI is formulated as:

$$\mu =\left\{\begin{array}{cc} V\cdot a\cdot \exp\left(RI\cdot b\right) & :a=1348,b=0.373\\ V\cdot a\cdot \exp\left(StdI\cdot b\right) & :a=1153,b=0.6446\\ V\cdot a\cdot \exp\left(EI\cdot b\right) & :a=0.2302,b=3.87 \end{array}\right.$$

**Figure 13:**
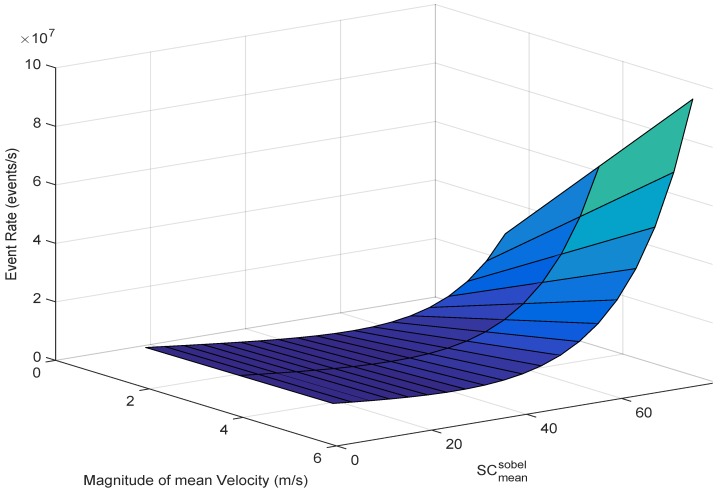
Event rate model as a function of the Sobel-based mean gradient and the magnitude of mean velocity.

Figure 13 shows the event rate as a function of the magnitude of mean velocity of the sensor and the Sobel-based mean gradient. In the following, we analyse the accuracy of the proposed model by utilizing the neuromorphic dataset [28].

### 4.3. Model Evaluation

In order to analyse the prediction accuracy of the model on unseen data, we extracted an evaluation dataset from [28], shown in Table 4, comprising both the indoor and outdoor scenes with different motions of the camera. The sequences belonging to the outdoor dataset were *Walking*, *Running* and *Urban*, whereas the remaining sequences belonged to the indoor dataset. The first column reports the name of the dataset along with the extracted time duration; for instance, the *Walking* dataset comprises a 33 second-long sequence from 100–133 s. The table reports scene complexity, in terms of Sobel-based mean gradient and magnitude of mean velocity. According to the table, the model showed good prediction accuracy for the outdoor environment. For instance, the average prediction accuracy of the outdoor environment was approximately 88.47%, whereas the average prediction accuracy for the indoor environment was approximately 79.60%. It is important to note that the indoor motion ranged from slider-based linear motion to the more complex three-dimensional translation motion in the X, Y and Z directions. The majority of the available dataset comprises a variable and more than one degree of freedom motion. Accordingly, the majority of the data utilized for deriving the model correspond to variable motion in the X, Y and Z directions. Therefore, the constant linear motion data (*Slider depth* and *Slider far*) resulted in lower prediction accuracy as compared to variable multi-direction motion data.

The model only considers the magnitude of the mean velocity and does not consider the direction of motion. We expected that this would increase the prediction accuracy at the expense of a considerable increase in the model complexity. However, the model still achieved an overall average prediction accuracy of 84% with a simple two-parameter single-term exponential model. Another important factor contributing to the prediction error is the noise, which generally originates from the bias settings of the sensor.

**Figure 14:**
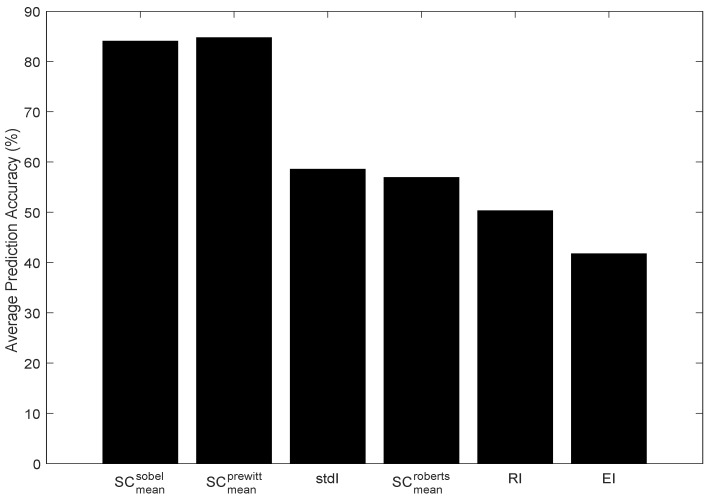
Average prediction accuracy of different models.

Figure 14 shows the prediction accuracy of all the models presented in Section 4.2. According to the figure, the models based on Sobel- and Prewitt-based mean gradient showed the best prediction accuracy. According to the results analysed in Section 3, mean gradients based on the Sobel and Prewitt operators achieved the lowest RMSE and the highest correlation performance with the neuromorphic event rate. On the other hand, the 2 × 2 Roberts cross operator achieved the lowest prediction accuracy amongst all the mean gradient based models. The small size of the Roberts operator caused the gradient magnitude to be sensitive to noise. This can cause genuine edges, which can be a major source of spike event generation, to be affected by noise. Figure 14 also reports the prediction accuracy of the metrics based on scene texture. According to the figure, the model based on entropy achieved the lowest prediction accuracy, i.e., approximately 42%. This is mainly because entropy is oblivious to the spatial structure present in the scene, whereas the neuromorphic rate of change of brightness is highly dependent on the spatial distribution of pixels. The other two texture-based models, stdI and RI, performed better than the entropy-based model.

#### Impact of the Moving Objects in a Scene

The outdoor sequences in the evaluation dataset, reported in Table 4, have moving objects in the scene. Our model did not explicitly consider the motion of objects in the scene and the relevant impact on the event rate; hence, we analysed the estimation error for sequences with moving objects in the scene. The outdoor sequences in the dataset are those characterized by higher motion in the scene. According to Table 4, the impact of moving objects was more pronounced when the speed of the sensor was low. For instance, the event rate prediction error in the outdoor *Urban* sequences was approximately 20%, where the velocity of the sensor was 0.65 m/s. This is mainly because of the proportion of events produced by the object movement. On the other hand, the increase in sensor speed decreased the percentage error. The outdoor *Walking* and *Running* sequences, where the sensor moves at a higher speed, resulted in a better prediction accuracy, mainly because the proportion of events produced by the sensor motion prevailed over the events produced due to the object movement. Therefore, the model was capable of achieving better prediction accuracy in scenarios where the velocity of the sensor was higher. The prediction accuracy of the model can be improved for low sensor speed scenarios by considering in the model the motion of objects in the scene.

## 5. Comparison of Neuromorphic Sensors and Conventional Ones for Sensor Network Devices

Sensor network devices have limited resources in terms of memory and processing power. Visual sensors with a low data rate consume less power in the device. Therefore, the source data rate becomes extremely important for resource-constrained sensor network devices. In this section, we compare the data rate of neuromorphic and conventional vision sensors by utilizing the evaluation dataset in the previous section.

Table 5 compares the data rate output by neuromorphic and conventional vision sensors. The transmission of raw video using a conventional vision sensor, at a resolution 240×180 and a frame rate of 1000 fps, requires a data rate of 43.2 MB/s (grey-scale only). The data rate reduces to 4.32 MB/s for 100 fps. To reduce the size of the transmitted data, a solution is to compress the data at the sensing device. Although raw frame-based video data could be compressed with existing compression technologies, existing compression algorithms for conventional vision sensors violate the memory and latency constraints of sensor network devices [37].

On the other hand, the DVS requires only a fraction of the bandwidth mainly because these sensors capture the dynamics of a scene, thus filtering out redundant information. For applications where a higher frame rate is not desired, the time-stamp information in the neuromorphic data can be filtered out to achieve a lower data rate. Such a filtering policy requires only a spatial location histogram map and location histogram counts [38]. The filtering policy further reduced the data rate, approximately seven-times the original one, at the cost of a lower frame rate of 100 fps, as shown in Table 5.

## 6. Conclusions

Event-based cameras are very promising sensors for high-speed mobile robots, self-driving cars and drones and many other applications ranging from next-generation Internet of Things to low-latency access to visual medical information. The data rate output by the event-based vision sensors depends on the type of scene and motion speed. In this work, we proposed a bandwidth prediction model by considering the scene content and motion speed. According to the study, the neuromorphic event rate varied exponentially with the scene complexity, with Sobel- and Prewitt-based mean gradient approximation showing the best goodness of fit. In addition, we studied the relationship between the sensor speed and the event rate, which we found to be linear. Based on these analyses, we proposed a two-parameter exponential model for the dependency of the event rate on scene complexity and sensor speed. According to the results, Sobel- and Prewitt-based models showed prediction accuracy of approximately 88.4% for the outdoor dataset along with the overall prediction performance of approximately 84%.

## Figures and Tables

**Table 1:** Goodness of fit statistics of the exponential model for the gradient-based metrics.

		Exponential Fit		
Metric	$R^{2}$	adj $R^{2}$	RMSE	SSE
$SC_{\mathrm{std}}^{\mathrm{Sobel}}$	0.2252	0.1391	7.54 × 10${}^{6}$	5.12 × 10${}^{14}$
$SC_{\mathrm{rms}}^{\mathrm{Sobel}}$	0.9769	0.9743	1.3 × 10${}^{6}$	1.53 × 10${}^{13}$
$SC_{\mathrm{mean}}^{\mathrm{Sobel}}$	0.9935	0.9927	6.93 × 10${}^{5}$	4.33 × 10${}^{12}$
$SC_{\mathrm{mean}}^{\mathrm{Prewitt}}$	0.9939	0.9933	6.67 × 10${}^{5}$	4.01 × 10${}^{12}$
$SC_{\mathrm{mean}}^{\mathrm{Roberts}}$	0.9866	0.9851	9.91 × 10${}^{5}$	8.84 × 10${}^{12}$

**Table 2:** Goodness of fit statistics of the exponential model between event rate and binary edge detection metrics.

		Exponential Fit		
Metric	$R^{2}$	adj $R^{2}$	RMSE	SSE
$EP^{Canny}$	0.981	0.979	1.182 × 10${}^{6}$	1.257 × 10${}^{13}$
$EP^{Sobel}$	0.01094	−0.09896	8.521 × 10${}^{6}$	6.535 × 10${}^{14}$
$EP^{Prewitt}$	0.01091	−0.09899	8.522 × 10${}^{6}$	6.535 × 10${}^{14}$
$EP^{Roberts}$	0.006121	−0.1043	8.542 × 10${}^{6}$	6.567 × 10${}^{14}$

**Table 3:** Goodness of fit statistics of the exponential model for the texture-based metrics.

		Exponential Fit		
Metric	$R^{2}$	adj $R^{2}$	RMSE	SSE
StdI	0.9889	0.9876	9.04 × 10${}^{5}$	7.35 × 10${}^{12}$
RI	0.9848	0.9831	1.06 × 10${}^{6}$	1.0 × 10${}^{13}$
EI	0.9663	0.9626	1.57 × 10${}^{6}$	2.22 × 10${}^{13}$

**Table 4:** Prediction accuracy of the Sobel-based mean gradient model on different outdoor and indoor datasets.

Extracted	Mean	Metric	Event Rate	Event Rate	Prediction
DAVIS	Velocity (*V*)	${\mathrm{SC}}_{\mathrm{mean}}^{\mathrm{Sobel}}$	Model	Camera	Accuracy
Dataset	(m/s)		(events/s)	(events/s)	
Walking (100–133.4)	1.42	33.06	$0.521\times 10^{6}$	$0.5333\times 10^{6}$	97.77%
Running (1–43.5)	3.1	29.9	$0.855\times 10^{6}$	$1.0202\times 10^{6}$	83.82%
Running (43.5–87)	3.1	35.13	$1.37\times 10^{6}$	$1.25\times 10^{6}$	90.4%
Running (1–87)	3.1	32.52	$1.084\times 10^{6}$	$1.13\times 10^{6}$	97.12%
Urban (1–5)	0.65	39.95	$0.445\times 10^{6}$	$0.535\times 10^{6}$	79.78%
Urban (5–10)	0.65	41.85	$0.529\times 10^{6}$	$0.625\times 10^{6}$	81.9%
Box (1–25)	0.57	50	$0.97\times 10^{6}$	$1.08\times 10^{6}$	89.81%
Slider far (1–6.4)	0.16	60	$0.673\times 10^{6}$	$0.538\times 10^{6}$	75.47%
Slider depth (1–3.2)	0.32	46.82	$0.408\times 10^{6}$	$0.3172\times 10^{6}$	71.4%
Calibration (1–10)	0.11	54.04	$0.27\times 10^{6}$	$0.349\times 10^{6}$	77.37%
Dynamic (1–30)	0.85	34.3	$0.349\times 10^{6}$	$0.433\times 10^{6}$	80.6%
Dynamic (30–59)	1.2	34.7	$0.511\times 10^{6}$	$0.598\times 10^{6}$	83%

**Table 5:** Bandwidth comparison between the DVS and the conventional vision sensor. The spatial resolution of 240×180 is considered for both types of sensors.

Extracted	Data Rate	Data Rate	Data Rate	Data Rate
DAVIS	Events Camera	Conventional Camera	Events Camera	Conventional Camera
Dataset	1000 fps (MB/s)	1000 fps (MB/s)	100 fps (MB/s)	100 fps (MB/s)
Walking (100–133.4)	1.066	43.2	0.152	4.32
Running (1–43.5)	2.04	43.2	0.255	4.32
Running (43.5–87)	2.5	43.2	0.334	4.32
Running (1–87)	2.26	43.2	0.322	4.32
Urban (1–5)	1.07	43.2	0.154	4.32
Urban (5–10)	1.25	43.2	0.176	4.32
Box (1–25)	2.16	43.2	0.27	4.32
Slider far (1–6.4)	1.076	43.2	0.154	4.32
Slider depth (1–3.2)	0.6344	43.2	0.09	4.32
Calibration (1–10)	0.698	43.2	0.107	4.32
Dynamic (1–30)	0.866	43.2	0.121	4.32
Dynamic (30–59)	1.196	43.2	0.171	4.32

## References

[1] Posch C., Benosman R., Cummings R.E. (2015). Giving Machines Humanlike Vision similar to our own would let devices capture images more efficiently. IEEE Spectr..

[2] Fukushima K., Yamaguchi Y., Yasuda M., Nagata S. (1970). An electronic model of the retina. Proc. IEEE.

[3] Mead C., Mahowald M. (1988). A silicon model of early visual processing. Proc. IEEE.

[4] Lichtsteiner P., Posch C., Delbruck T. (2008). A 128 x 128 120 dB 15 *μ*s Latency Asynchronous Temporal Contrast Vision Sensor. IEEE J. Solid-State Circuits.

[5] Lichtsteiner P., Posch C., Delbruck T. A 128 × 128 120 dB 30 mW asynchronous vision sensor that responds to relative intensity change. Proceedings of the IEEE International Solid-State Circuits Conference (ISSCC).

[6] Mueggler E., Forster C., Baumli N., Gallego G., Scaramuzza D. Lifetime estimation of events from Dynamic Vision Sensors. Proceedings of the IEEE International Conference on Robotics and Automation (ICRA).

[7] Brandli C., Berner R., Yang M., Liu S., Delbruck T. (2014). A 240 × 180 130 dB 3 *μ*s Latency Global Shutter Spatiotemporal Vision Sensor. IEEE J. Solid-State Circuits.

[8] Sivilotti M. (1991). Wiring Consideration in Analog VLSI Systems with Application to Field Programmable Networks. Ph.D. Thesis.

[9] Barrios-Avilés J., Rosado-Muñoz A., Medus L.D., Bataller-Mompeán M., Guerrero-Martínez J.F. (2018). Less Data Same Information for Event-Based Sensors: A Bioinspired Filtering and Data Reduction Algorithm. Sensors.

[10] Chikhman V., Bondarko V., Danilova M., Goluzina A., Shelepin Y. (2012). Complexity of images: Experimental and computational estimates compared. Perception.

[11] Cilibrasi R., Vitanyi P.M.B. (2005). Clustering by compression. IEEE Trans. Inf. Theory.

[12] Yu H., Winkler S. Image complexity and spatial information. Proceedings of the IEEE International Conference on Quality of Multimedia Experience (QoMEX).

[13] ANSI T1.801.03 (1996). Digital Transport of One-Way Video Signals—Parameters for Objective Performance Assessment.

[14] Cover T.M., Thomas J.A. (2016). Elements of Information Theory.

[15] Li M., Chen X., Li X., Ma B., Vitanyi P.M.B. (2004). The similarity metric. IEEE Trans. Inf. Theory.

[16] Cardaci M., Gesù V.D., Petrou M., Tabacchi M.E. (2009). A fuzzy approach to the evaluation of image complexity. Fuzzy Sets Syst..

[17] Perkio J., Hyvarinen A. Modeling image complexity by independent component analysis, with application to content-based image retrieval. Proceedings of the International Conference on Artificial Neural Networks (ICANN).

[18] Romero J., Machado P., Carballal A., Santos A. (2012). Using complexity estimates in aesthetic image classification. J. Math. Arts.

[19] Tedaldi D., Gallego G., Mueggler E., Scaramuzza D. Feature Detection and Tracking with the Dynamic and Active-Pixel Vision Sensor. Proceedings of the IEEE International Conference on Event-Based Control, Communication, and Signal Processing (EBCCSP).

[20] Rigi A., Baghaei Naeini F., Makris D., Zweiri Y. (2018). A Novel Event-Based Incipient Slip Detection Using Dynamic Active-Pixel Vision Sensor (DAVIS). Sensors.

[21] Maqueda A.I., Loquercio A., Gallego G., Garcia N., Scaramuzza D. Event-Based Vision meets Deep Learning on Steering Prediction for Self-Driving Cars. Proceedings of the IEEE Conference on Computer Vision and Pattern Recognition (CVPR).

[22] Mueggler E., Huber B., Scaramuzza D. Event-Based, 6-DOF Pose Tracking for High-Speed Maneuvers. Proceedings of the IEEE International Conference on Intelligent Robots and Systems (IROS).

[23] Khan N., Martini M.G., Staehle D. (2014). QoS-aware composite scheduling using fuzzy proactive and reactive controllers. J. Wireless Com Network.

[24] Khan N., Martini M.G., Staehle D. Opportunistic QoS-Aware Fair Downlink Scheduling for Delay Sensitive Applications Using Fuzzy Reactive and Proactive Controllers. Proceedings of the IEEE Vehicular Technology Conference (VTC).

[25] Nasralla M.M., Khan N., Martini M.G. (2018). Content-aware downlink scheduling for LTE wireless systems: A survey and performance comparison of key approaches. Comput. Commun.

[26] Khan N., Martini M.G. (2016). QoE-driven multi-user scheduling and rate adaptation with reduced cross-layer signaling for scalable video streaming over LTE wireless systems. J. Wireless Com Network.

[27] Khan N., Martini M.G. Data rate estimation based on scene complexity for dynamic vision sensors on unmanned vehicles. Proceedings of the IEEE International Symposium on Personal, Indoor and Mobile Radio Communications (PIMRC).

[28] Mueggler E., Rebecq H., Gallego G., Delbruck T., Scaramuzza D. (2017). The Event-Camera Dataset and Simulator: Event-Based Data for Pose Estimation, Visual Odometry, and SLAM. Int. J. Robot. Res..

[29] Sobel I., Feldman G., Duda R., Hart P. (1968). A 3 x 3 isotropic gradient operator for image processing, presented at a talk at the Stanford Artificial Project. Pattern Classification and Scene Analysis.

[30] Prewitt J.M.S., Lipkin B., Rosenfeld A. (1970). Object enhancement and extraction. Picture Processing and Psychopictorics.

[31] Roberts L.G. (1963). Machine Perception of Three-Dimensional Solids. Ph.D. Thesis.

[32] Barman N., Martini M.G. H.264/MPEG-AVC, H.265/MPEGHEVC and VP9 codec comparison for live gaming video streaming. Proceedings of the IEEE International Conference on Quality of Multimedia Experience (QoMEX).

[33] Barman N., Zadtootaghaj S., Martini M.G., Moller S., Lee S. A Comparative Quality Assessment Study for Gaming and Non-Gaming Videos. Proceedings of the IEEE International Conference on Quality of Multimedia Experience (QoMEX).

[34] Haseeb A., Martini M.G. Rate and distortion modeling for real-time MGS coding and adaptation. Proceedings of the IEEE Wireless Advanced Conference (WiAd).

[35] Parker J.R. (1996). Algorithms for Image Processing and Computer Vision.

[36] Canny J.F. (1986). A computational approach to edge detection. IEEE Trans. Pattern Anal. Mach. Intell..

[37] Yap F.G.H., Yen H.H. (2014). A Survey on Sensor Coverage and Visual Data Capturing/Processing/Transmission in Wireless Visual Sensor Networks. Sensors.

[38] Bi Z., Dong S., Tian Y., Huang T. Spike Coding for Dynamic Vision Sensors. Proceedings of the IEEE Data Compression Conference (DCC).

